# Immediate Effects of Mindful Awareness in Body-oriented Therapy as an Adjunct to Medication for Opioid Use Disorder

**DOI:** 10.21203/rs.3.rs-4727162/v1

**Published:** 2024-07-15

**Authors:** Cynthia U. Price, Kenneth C. Pike, Anna Treadway, Julia Palmer, Joseph O. Merrill

**Affiliations:** University of Washington

**Keywords:** opioid use disorder, intervention, mindfulness, randomized controlled trial, substance use disorder, interoception, mixed methods

## Abstract

**Objective:**

While effective, medication for opioid use disorder (MOUD) treatment outcomes can be limited by co-occurring polysubstance use, mental health and chronic pain conditions. Interoceptive training may facilitate well-being and support medication treatment for MOUD. This study examined the pre-post effects of the mindfulness-based intervention Mindful Awareness in Body-oriented Therapy (MABT) as an adjunct to MOUD. MABT teaches interoceptive awareness skills to promote self-care and emotion regulation.

**Methods:**

People stabilized on medication for OUD (N = 303) from 6 community clinics in Northwestern United States were recruited and randomly assigned to MABT plus MOUD or MOUD only. In a mixed-methods study, we used an intent-to-treat approach to examine the proportion of days abstinent from non-prescribed opioids, and other substance use (primary outcomes) at baseline and 3 months post-intervention. Secondary outcomes included symptoms of mental health distress; emotional regulation difficulties; pain and physical symptom indicators; interoceptive awareness and mindfulness skills. Participant experience of MABT was collected through post-intervention surveys. Changes in outcomes were assessed using linear mixed models; content analysis was used to analyze the qualitative data.

**Results:**

Levels of overall substance use were low and did not differ between groups. Significant improvements in PTSD symptoms, interoceptive awareness, pain severity, pain activity interference, and physical symptom frequency were found for those who received MABT compared to MOUD only.

**Conclusion:**

In this stable MOUD population, substance use outcomes were not improved, however MABT demonstrated significant positive changes across multiple health outcomes critical for improving MOUD treatment. Clinical Trials Registration: NCT04082637 on 9/3/2019

## Introduction

The epidemic of opioid use disorder and overdose death has led to the expansion of MOUD through multiple state and federal initiatives, raising the question of whether adjuncts to opioid use disorder treatment can be developed to improve outcomes compared with standard medical management. In 2019, approximately 1.6 million Americans ages 12 and over had opioid use disorder (OUD) (Center for Behavioral Health Statistics and Quality, 2020). The most effective intervention shown to reduce drug use, morbidity, and mortality related to opioid use disorder is medication for opioid use disorder (MOUD) with buprenorphine or methadone, and potentially naltrexone (Ajazi et al., 2022; Larochelle et al., 2018a; Sordo et al., 2017). That said, the high prevalence of co-occurring poly-substance use (Mahoney et al., 2021), psychiatric disorders and chronic pain (Barry et al., 2016; Hser et al., 2017; Leyde et al., 2024) among those with OUD are associated with poor treatment outcomes, including drug use and treatment retention, in this population (Higgins et al., 2020; Rosic et al., 2017; Tsui et al., 2020). The need for behavioral strategies as adjunctive treatment support for this population to address the high levels of psychological and physical distress and pain is well-recognized (Jones & McCance-Katz, 2019; Novak et al., 2019). State and federal resources to expand medication treatment for opioid use disorder have had significant impact on the settings where treatment is available (SAMSHA, 2020; U.S. Department of Health & Human Services, 2018), with some medical settings developing systems of care that allow large numbers of patients to be treated without limitations on treatment duration (LaBelle et al., 2016; Wartko et al., 2023). This integration of OUD treatment services into chronic care settings makes urgent the development of behavioral treatment adjuncts to MOUD treatment that can address the ongoing and co-occurring issues common among those with OUD.

While there is some controversy about the effectiveness of psychosocial interventions for patients treated with medication for opioid use disorder (Dugosh et al., 2016; Schwartz, 2016; Trafton et al., 2004), the most robust trials have not shown a significant advantage of more intensive psychosocial interventions compared with medication management alone (Fiellin et al., 2013; Weiss, 2011). Mindfulness-based interventions (MBIs) differ from conventional psychosocial approaches in their focus on developing skills of present-moment awareness to facilitate self-awareness, acceptance, and self-compassion of internal experiences and reactions to external circumstances, and to recognize these as transient experiences (Kabat-Zinn, 2003). Developing these skills can lead to insight and positive shifts in automatic reactions and behaviors that are critical for shifting emotional response patterns that can lead to substance use and relapse (Appel & Kim-Appel, 2009; Segal et al., 2002). MBIs show promising treatment effects when delivered within the context of substance use disorder (SUD) treatment, likely due to improving cognitive and affective processes underlying substance use (Priddy et al., 2018). Prior randomized controlled trials of MBI approaches for MOUD with published outcomes involve Mindfulness-Oriented Recovery Enhancement (MORE). MORE studies have demonstrated improvements in pain, opioid use, and other outcomes in chronic pain patients receiving methadone maintenance treatment, primary care patients with opioid misuse, and people with chronic pain on prescribed opioids (Cooperman et al., 2023; Garland et al., 2022; Garland et al., 2023; Garland et al., 2024).

The question has been raised about the potential role of gender in response to MBIs for SUD, due to known gender differences such as women having more mood disorders before developing an SUD compared to men (de Graaf et al. 2002), more co-occurring affective disorders after developing a SUD (Zilberman et al. 2003), and more lifetime exposure to sexual trauma (Santo et al., 2021; Rodriguez, et al, 2024). A review of MBIs for SUD treatment did not show a difference on outcomes due to gender (Katz & Toner, 2013), nor did a recent meta-analysis of MORE trials (Parisi, A., Roberts, L, Hanley, A., & Garland, E. (2022). However, one study examining gender differences in response to a MBI among college students (N=77) showed greater decreases in negative affect and greater increases in mindfulness domains of non-reactivity, non-judgement and observing emotions among women compared to men, suggesting that women and men may have different underlying emotional regulation processes that impact the response to mindfulness training (Rojiani et al., 2017). MBI studies have not typically examined the role of gender on health outcomes, a recommendation for future research (Katz & Toner, 2013).

One identified potential underlying mechanism for improved health outcomes among those in SUD treatment that is linked to regulation, is interoceptive sensibility (Garland, 2016; Witkiewitz et al., 2013), which is defined as the process of sensing, representing, and appraising the body’s internal state (Craig, 2003). Brain imaging studies demonstrate that those with high levels of symptomatic distress - including substance use disorder, mental health disorders, and chronic pain have impaired sensory processing compared to healthy controls (Quadt et al., 2018). A recent pilot fMRI study among women in SUD treatment found a positive association between an emotion regulation challenge task, dispositional mindfulness, and brain regions thought to be important for inhibitory control (Droutman et al., 2022). Various neurocognitive models and clinical research link interoception to regulation and health outcomes (Weng et al., 2021), suggesting the importance of interoceptive training to enhance emotional and physical well-being and regulation, and the potential of interoceptive training as an integrative approach to address the complex and co-occurring conditions so often present among those in MOUD.

Mindful Awareness in Body-oriented Therapy (MABT) targets the development of interoceptive sensibility to support self-awareness, regulation and overall mental and physical well-being (Price & Hooven, 2018). With its novel approach combining psychoeducation, touch-based coaching, and mindfulness to develop interoceptive awareness, MABT has shown promising results as an adjunct to intensive outpatient SUD treatment (Price et al., 2018, 2019; Price, Wells, Donovan, & Brooks, 2012; Price, Wells, Donovan, & Rue, 2012). Touch-based coaching involves manual touch by the therapist to guide client/participant attention to specific regions of the body to develop interoceptive awareness skills. The prior MABT studies in SUD treatment have been specifically for women in abstinent-based programs and have demonstrated significant improvement in interoceptive awareness and mindfulness skills, increased heart rate variability, and reductions in substance use and craving, mental health distress, emotion regulation difficulties, and frequency of physical symptoms. A recent neuroimaging study shows that increases in interoceptive sensibility (as measured on the MAIA) in response to MABT were associated with increased sensory processing, indicating brain plasticity and the potential to develop and change interoceptive capacity in response to interoceptive training (Price, Sevinc & Farb, 2023).

Core tenants of mindfulness are integral to MABT and a requisite skill for developing sustained interoceptive attention (Price & Weng, 2021). MABT differs from most MBIs in that it is delivered individually (vs. in a group), is focused on developing the capacity for sustained interoceptive attention, and the concomitant capacity for somatic reappraisal to promote regulation and integration of interoceptive skills into daily life to enhance self-care (Price and Hooven, 2017) (see description of MABT below for more information).

Except for a small initial pilot (Price et al., 2020), the current study was the first RCT to examine MABT as an adjunct to MOUD, and the first to examine MABT as adjunct to SUD for men and women. The purpose of this study was to compare those who received MABT + MOUD to those who received MOUD alone on health outcomes at baseline and three-month follow-up, and to triangulate these findings with qualitative data from those who received MABT to better understand intervention experience and related health benefits. The primary outcome was opioid use and overall substance use. The secondary outcomes examine change on multiple mental and physical health indicators. We hypothesized that MABT + MOUD compared to MOUD would result in (1) a higher percentage of days abstinent from opioids and overall substance use, (2) improved health outcomes including mental health distress; difficulties in emotion regulation; physical pain, pain interference and symptom frequency; interoceptive sensibility and mindfulness skills; and opioid craving. Given that there is some question regarding whether response to MBIs may differ by gender, and that this was the first MABT study to include men when delivered as an adjunct to SUD treatment, we explored the role of gender on health outcomes. In addition, this study reports on the themes that emerged regarding participant experience and perceived impact of the MABT intervention.

## Methods

### Participants

Participants were recruited from six outpatient MOUD clinics located in western Washington State. They attended clinics that varied in that they were in different regions of western Washington: three within a major metropolitan area, one in the mid-sized city, and one in a small city serving a largely rural community. The clinics were housed in different types of clinical settings (e.g. primary care, mental health, or substance use only). Five out of six prescribed buprenorphine and one dispensed methadone for MOUD, with all sites confirming an opioid use disorder diagnosis at treatment entry.

Potential participants were referred to the study by clinic staff (i.e. nurses, physicians and counselors), with confirmation of an active OUD diagnosis. The clinics varied in that they were in different regions of western Washington: three within a major metropolitan area, one in the mid-sized city, and one in a small city serving a largely rural community. The clinics were housed in different types of clinical settings (e.g. primary care, mental health, or substance use only). Five out of six prescribed buprenorphine and one dispensed methadone for MOUD, with all sites confirming an opioid use disorder diagnosis at treatment entry.

The Research Coordinator associated with each clinical site screened prospective participants by phone for study eligibility after describing the study in detail. Inclusion criteria were: 1) diagnosed with OUD; 2) enrolled in a medication treatment program for opioid use disorder; 3) over 18 years old; 4) stable on medication dose, involving (if buprenorphine) at least four weeks of medication treatment and appointment frequency of less than once/week (to ensure completed initiation and attained degree of stability); involving (if methadone) at least 90 days in treatment with a minimum dose of 60mg, no missed dose evaluation appointments in past 30 days, and no more than 3 missed doses in 30 days; 5) willing to forego (non-study) manual (e.g., massage) and/or mind-body therapies (e.g., mindfulness meditation) for 3 months (baseline to post-test); 6) willing to sign release for access of electronic medical records; 7) fluent in English; 8) able to attend study sessions when offered. Exclusion criteria: 1) unwilling or unable to remain in MOUD treatment for the duration of the trial (includes planned relocation, pending extended incarceration, etc); 2) over 24 weeks gestation, if pregnant, to avoid intervention interruptions related to childbirth; 3) unmedicated psychosis or other conditions such as cognitive impairment, to be assessed with an adapted 7-item Mini-Mental Status Exam (MMSE), [36] if there was reported brain injury or questionable comprehension of the consent form.

**Figure 1:**
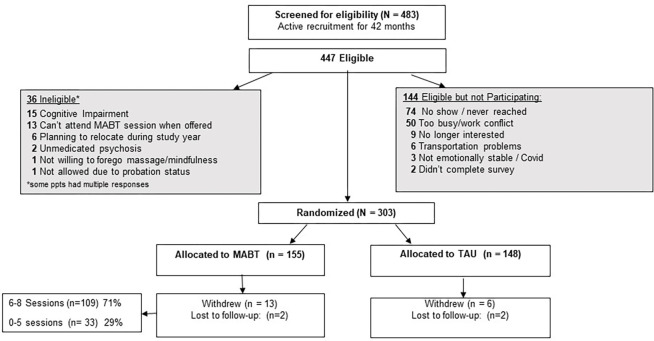
Flow Diagram

Four hundred eighty-three individuals in MOUD treatment expressed interest in the study and were screened for participation between August 2019 and January 2023. Of those screened, 447 (~ 92%) met study eligibility criteria, and among these, 144 (32%) did not participate. Primary reasons for non-participation were being unreachable or not showing up for baseline appointment and indicating that they were too busy to participate (see Fig. 1). In total, 303 individuals enrolled in the study and were randomly assigned to one of the two study groups. No one declined study involvement due to randomization assignment. Approximately 6% of the enrolled participants withdrew from study participation soon after enrollment, and another 8% were lost to follow-up and did not complete an assessment after baseline. The two primary reasons given for not participating in the study by those who formally withdrew were a lack of time or lack of interest. Of the participants assigned to MABT, 109 (71%) completed the intervention program, defined as completing at least 75% of the intervention (equivalent to 6 or more of the 8 sessions); involving exposure to all stages of the protocol, (see Fig1).

Sample demographics and clinical characteristics are summarized in Table 2. Ages ranged from 21–73 with a median of 40 years of age, and there were almost equivalent numbers of people who identified as men (48%) and women (52%); two people identified as non-binary. Cultural and racial identity matches the region, with 9% Hispanic, and racial identifies as White (79%), 9% as Mixed-Race, 5 % as Black, 4% as Native American, 1% as Asian and 1% as Native Hawaiian or Pacific Islander. Socioeconomic status was low with 35% employed (at either full or half time), about 60% reporting less than $1000/month in income, the majority (72%) on Medicaid for health insurance, and 56% reporting at most a high school education. Prior to study enrollment the majority (67%) had been in MOUD treatment for over 12 months. Fifty-seven percent of the sample met the criteria for chronic pain, and having chronic pain was associated with higher mental health symptoms (Leyde et al., 2024). Mental health distress was high with 41% screening above the screening cut-off for PTSD, 49% above the cut-off for moderate depression, and 40% above the cut-off for moderate anxiety. There was remarkable exposure to traumatic events across the lifespan with over 50% of the sample reporting sexual abuse during childhood, adult physical assault (by a stranger), intimate partner violence, and exposure to traumatic accidents (for more details on lifetime trauma in this sample, see Rodriguez et al., 2024). Of note, less than half (47%) reported seeing a mental health professional in the past 90 days.

### Procedure

Those eligible who expressed interest were scheduled for an appointment to complete informed consent documents, baseline assessment, and randomization to either the study intervention as an adjunct to usual care (MABT + MOUD) or usual care/control arm (MOUD). Study involvement was for one year, with assessments delivered at five timepoints: baseline, 3, 6, 9 and 12 months. This study presents findings from the intervention period from baseline to 3-month assessment. A future publication will present the longitudinal results, for which the project was powered, based on longer term substance use outcomes.

Randomization was specific to each study site and stratified by gender (approximately equal numbers of men and women assigned to each study group at each site) and self-report (yes/no) of chronic pain, defined as self-report of ongoing pain for at least three months duration. Participants are randomized via the software program Rand.F ‘(Cain & Rand, 2009) using an algorithm that is a modification of the minimization method (Pocock, 1983). Participants assigned to MABT were immediately put in touch with the MABT therapist at the clinic to schedule weekly MABT sessions. The MABT intervention was delivered in the period between baseline and 3-month assessment, by a licensed massage therapist trained in the MABT approach. There were 1 or 2 MABT therapists working at each clinic, depending on the clinic size and anticipated study enrollment numbers.

All study procedures occurred at the clinic where the participant received MOUD. Participants were remunerated $30 for completing the baseline assessment, $40 for completing the 3-month assessment; for those assigned to MABT there was a $10 gift card given after completion of each MABT session. To assist with transportation, if needed, we gave bus passes or a $5 gift card to go toward gas money.

The COVID pandemic took place during this study (data collection: August 2019 – January 2024). Study procedures remained the same throughout the COVID pandemic, with the exception of a 5-month pause in enrollment and pause in intervention delivery (March – July 2020), in compliance with WA State and University of WA mandates. The overall impact of this pause was relatively minimal as only two participants (newly assigned to MABT) were not able to attend sessions, and all other participants actively engaged in MABT were able to complete their intervention sessions prior to the pause. Follow-up assessments during this period were delivered remotely, mostly via zoom. After the 5-month enrollment/intervention pause, protective measures were put into place to mitigate COVID exposure; we then resumed study enrollment, in-person assessments, and intervention delivery.

#### MOUD – Usual Care Control Condition

All participants were receiving ongoing MOUD treatment as part of clinical care, which served as the “treatment as usual” control comparison condition. At each clinic, routine initial intake in MOUD involved a comprehensive assessment of substance use and related consequences, medical and mental health status, and current barriers to and supports for recovery; formal diagnosis of opioid use disorder and appropriateness for MOUD; scheduling and monitoring of medication initiation and urine drug testing. Limited counseling and/or behavioral health services were available at all clinical settings but required only for those receiving methadone. Best practice in MOUD is to continue seeing patients if drug use is maintained or there is relapse, due to evidence of MOUD benefits and the potential life-saving effects. Given this, no site discontinued MOUD in response to a participant’s return to drug use.

The COVID pandemic changed policies and procedures at some clinical sites for approximately 1 year of the study period (starting in March 2020), primarily reducing urine drug testing and use of telemedicine instead of in-person visits in buprenorphine settings and increased take-home methadone dosing in the opioid treatment program.

#### Mindful Awareness in Body-oriented Therapy (MABT) + MOUD – Intervention Condition

The MABT intervention has a well-developed protocol and training manual for research, and is delivered individually in 1.25-hour sessions, once/week for 8 weeks as an adjunct to MOUD. All MABT sessions had to be completed prior to the 3-month assessment. The intervention was designed to sequentially teach sensory awareness and mindfulness skills to build interoceptive capacity and includes weekly take-home assignments for practicing and incorporating MABT skills into daily life (Price & Hooven, 2018). The program is organized into three distinct stages (See Table 2). Stage 1 (sessions 1–2), develops body literacy, the ability to identify and articulate sensory awareness. Stage 2 (sessions 3–4) focuses on interoceptive awareness training, to access inner body awareness and begin to make links between physical and emotional sensations. Stage 3 (sessions 5–8) furthers the development and practice of mindful body awareness, involving sustained mindful presence with interoceptive attention on regions of the body, and aims to facilitate positive shifts in sensory experience as well as insights that promote somatic reappraisal and support behavior change (Price & Weng, 2021). The Intake and Session Review aspects of the session are critical. Intake, taking approximately 20 minutes at the beginning of each session, is designed to gather information about the participant’s emotional and physical well-being, describe MABT processes, discuss use of take-home practice, and develop rapport. At the end of each session, approximately 15 minutes is reserved to review the interoceptive awareness training experience to promote cognitive integration and somatic reappraisal that then guides the collaborative development of a take-home practice for the interim week.

MABT, which integrates the use of touch to help orient and maintain mindful attention to the body (vs. having attention wander, for example) when learning interoceptive skills, was delivered by licensed and experienced massage therapists who were trained in the MABT protocol. The MABT approach involves a high level of client-therapist verbal interaction throughout the sessions. Thus, all MABT therapists (one or two at each of the 6 clinics depending on clinic size and anticipated recruitment) had considerable prior education and clinical experience, including advanced training and/or certification in mind-body or psychotherapy approaches (e.g. Hakomi, Mindfulness Meditation, Focusing) and some of the therapists were dually licensed as Masters-level mental health therapists. A MABT therapist and trainer was employed on the research team to monitor intervention fidelity and provide clinical supervision for therapists on a weekly basis. All MABT sessions were digitally recorded. Implementation fidelity monitoring included weekly review of audio-recorded sessions, and process evaluation forms completed by study therapists after each MABT session. In addition, MABT therapists received weekly clinical supervision.

### Measures

The same set of outcome measures were administered at both pre and post assessment timepoints with the exception of three questionnaires included only at baseline: a demographic and health history questionnaire (adapted from the Addiction Severity Index), (McLellan et al., 1992) and the Trauma Lifetime Events Questionnaire (TLEQ) (Kubany et al., 2000). For the follow-up assessment, there was a window of 6 weeks during which participants could attend the assessment visit to complete measures (2 weeks prior to 4 weeks after the 3-month date of their baseline appointment).

In addition to the assessment procedures outlined above, we collected data from electronic medical records specific to time in MOUD treatment prior to enrollment and participant self-report of mental health services received as a supplement to MOUD. From participants we collected any adverse events during study involvement, as well as reported practice of MABT skills during the intervention period from participants assigned to MABT.

#### Substance Use

Substance use was the primary measure and was assessed using the Time-Line Follow-back interview (TLFB), which has demonstrated validity (Sobell & Sobell, 1992; Sobell LC et al., 1996) to assess use of alcohol, non-prescribed or illicit drug use, and use of marijuana (which is typically not prescribed and legal in WA state) over the past 90 days. The primary outcomes were a) the percent days abstinent from non-prescribed opioid use and b) the percent days abstinent from overall substance use, including heavy drinking (≥ 4 drinks for a woman, ≥5 drinks for a man in a day) and non-prescribed drugs (with the exception of marijuana). The baseline TLFB included the 90-day period prior to entering the study. The subsequent TLFB was based on the number of days in the assessment period from baseline to the 3-month assessment (approximately 90 days).

#### Mental Health Distress

To measure mental health distress, we used three well-validated scales, each of which has clinical cut-off scores for diagnostic screening: the 20-item, 5-point likert-type scale Posttraumatic Stress Disorder (PTSD) Checklist (PCL-5) (Dickstein et al., 2015); the 9-item, 4-point likert-type scale Patient Health Questionnaire (PHQ-9) for depression (Kroenke et al., 2001); and the 7-item, 4-point likert-type scale General Anxiety Disorder screening (GAD-7) (Spitzer et al., 2006). In this sample the internal consistency reliability (Cronbach’s alpha/McDonald’s omega) for each of these scales was: 0.93/0.93 for the PCL-5, 0.86/0.86 for the PHQ-9, and 0.91/0.91 for the GAD-7.

#### Emotion Relation Difficulties

We used the 18-item, 5-point likert-type scale Difficulties with Emotion Regulation Short Form (DERS-SF) (Kaufman et al., 2016). Six subscales assess nonacceptance, goal-directed behavior, impulse control, awareness, regulation strategies, and emotional clarity. Higher scores correspond to more difficulties in emotion regulation. In this sample, the Cronbach’s alpha/McDonald’s omega were identical for the DERS; DERS Total was 0.90, and the subscales ranged from 0.75 – 0.86.

#### Pain Severity, Pain Interference, and Physical Symptoms

The Brief Pain Inventory, a well-validated and reliable pain scale (Poquet & Lin, 2016), was used to measure pain severity and pain interference. We used the mean score of the pain intensity items to assess pain severity, and two interference scales that represent distinct interference dimensions (Cleeland et al., 1996; Miettinen et al., 2019): activity interference (walking, work, general activity) and affective interference (mood, relation with others, enjoyment of life, sleep). National guidelines regarding pain measures in clinical trials identify a minimally important difference on the pain interference scale and is based on a decrease of 1 point (Dworkin et al., 2008; Farrar, 2010). In this sample, the Cronbach’s alpha/McDonald’s omega for the BPI Severity measure was 0.88/0.89; 0.89/0.90 for affective interference and 0.89/0.90 for activity interference.

Physical Symptoms were assessed through the Medical Symptoms Checklist (Leserman al., 1998), which measures the number and frequency of 33 common physical symptoms. The score was based on the mean frequency of endorsed symptoms assessed using a 5-point scale from “never to “always or almost always.” The Cronbach’s alpha/McDonald’s omega in this sample was 0.90/0.91.

#### Interoceptive and Mindfulness Skills

Interoceptive sensibility was assessed using the 37-item, 6-point likert-type version 2 of the Multidimensional Assessment of Interoceptive Awareness scale (Mehling et al., 2012, 2018). A well validated and reliable measure, the MAIA has 8 distinct scales that ask the frequency from “never” to “always” of practicing interoceptive skills; higher scores are thought to indicate more adaptive body awareness. The eight scales are organized into five domains: 1) General Awareness of Body Sensations is measured via the *Noticing* scale; 2) Emotional and Attentional Responses to Bodily Discomfort or Pain is measured via the *Not-Worrying* and the *Not-Distracting* subscales; 3) Attention Regulation (which captures the tendency to maintain and regulate attention to body sensations; 4) Awareness of Mind-Body Integration includes the *Emotional Awareness, Self-Regulation* and *Body Listening* scales. *Emotional Awareness* refers to consciousness of the interrelation of emotions and body sensations. *Self-Regulation* refers to the ability to control psychological distress by consciously attending to body sensations. *Body Listening* refers to active listening to the body for insight; 5) Tendency to trust body sensations is measured by the *Trusting* scale. The Cronbach’s alpha and McDonald’s omega were identical for the MAIA Total and individual scales: 0.90 for MAIA Total and a range from 0.81–0.90 for the individual scales except for 0.72 for the *Not-Worrying* scale.

We used the 14-item, 4-point likert-type Freiburg Mindfulness Inventory (Walach et al., 2006) to measure mindfulness skills. This scale is oriented toward fundamental mindfulness characteristics of openness, acceptance, curiosity and presence. The Cronbach’s alpha/McDonald’s omega was 0.89/0.90.

#### Opioid craving

Opioid craving level over the past week was assessed for: a) the medication prescribed to treat OUD (i.e. buprenorphine or methadone) for the full sample using a single-item on an 11-point numeric scale, with “0” being “no craving” and “10” being “strongest craving ever” (Rosenberg, 2009). In addition, we asked participants who endorsed craving for either a) prescribed opioids or b) any non-prescribed opioid (i.e. heroin, oxycontin, etc) to rate their level of craving on a 1–10 point numeric scale.

#### Intervention experience

The MABT Intervention Qualitative Survey was delivered at post-test to only those who were assigned to MABT. This survey asked a series of open-ended questions about participant experience, learned skills, and the perceived impact of MABT on their MOUD treatment.

### Data Analyses

#### Statistical Analysis

Descriptive statistics, including measures of central tendency (mean, median) and variability (e.g. standard deviation) were used. Baseline group equivalence was examined using T-tests and chi square tests and the study groups were found to be equivalent on demographic (e.g., age, gender, race, education) and outcome variables. T-tests and chi square tests were also used to explore possible baseline differences among those that completed the intervention compared to those who did not, and none were found.

In addition, we evaluated baseline equivalence by clinical site, medication type (buprenorphine vs methadone), and chronic pain status using one-way ANOVAs. Site differences were found on substance use and mental health distress (i.e. symptoms of depression, anxiety, PTSD). These same baseline health outcomes were similarly different when we compared clinics based on whether they served a primarily urban or rural population. The data showed that the two clinics driving the differences for both comparisons above were the two urban clinics that served more highly distressed patients: one provided buprenorphine and the other methadone. We then compared these two clinics on baseline outcomes using t-tests to determine whether medication type (i.e. methadone vs buprenorphine) might explain the difference but found these clinical sites to be equivalent on baseline outcome variables.

We then evaluated equivalence on baseline measures between those with and without chronic pain using t-tests, since approximately 50% of our sample endorsed chronic pain. We found higher mental health distress (anxiety, depression and PTSD symptoms) as well as an expected higher level of pain severity and interference at baseline among those with chronic pain compared to those without chronic pain (Leyde et al., 2024).

To control for the various baseline differences on outcomes between treatment groups in this sample, we used entropy balancing (Hainmueller, 2012; Hainmueller & Xu, 2013). Entropy balancing created matching weights for the treatment and control groups on demographic and outcome variables, including site, time in treatment prior to enrollment, chronic pain status, levels of mental health distress, levels of pain severity and interference, gender and age. While entropy balancing is a strategy typically used in observational studies, this matching procedure allowed us to run the main analysis without the use of multiple covariates and to retain the full sample in the analysis (rather than, for example, splitting out the sample based on chronic pain status).

Analysis of all outcomes utilized linear mixed models. For the primary outcomes of substance use we used mixed multilevel models (Stata 18.0, Mixed procedure) to test for study group differences across time. This longitudinal model included terms for the effect of group (MABT vs MOUD), month (0 vs 3), gender, month × group × gender interaction and a covariate, number of days in the assessment period (to account for any variation in the assessment period among participants (e.g. mean assessment period was 91.5 days with a standard deviation of 10.6) as fixed effects and a random intercept to account for within-subject correlations. Models included entropy balancing weights and were estimated using robust standard errors. For the secondary outcomes we used a similar approach but these models did not include a covariate for number of days in the assessment period. Due to there being no difference between treatment groups in mental health services reported at baseline or 3 three months, this variable was not included in the analysis. When the three-way interaction (group × time × gender) was not significant for any outcome, we reported only the group × time interaction. Group differences for both primary and secondary outcomes were assessed using Wald χ^2^ with significance set by a two-sided *P* < 0.05.

To test our expectations that the entropy matching approach would adequately control for the primary baseline differences of site and chronic pain status, we ran two additional analyses: the first was a linear mixed model with site as a covariate and we found the results were equivalent to the model with no covariate for site. The second was a sensitivity analysis with the chronic pain only subsample and the results were comparable to the main analysis with the full sample. These analyses confirmed that entropy balancing successfully controlled for these baseline differences.

#### Qualitative Analysis

A thematic analysis approach (Braun & Clarke, 2006) was used to identify themes emerging from post-intervention surveys documenting patient experiences with MABT. Using Atlas TI Version 23.2.3 (ATLAS.ti Mac, 2023), two research team members and study authors (AT and JKP) first separately coded the surveys to identify common themes for code book development. This was an iterative process and included input from the PI (CP). The second step involved separately categorizing responses in Atlas based on the identified codes. For any codes that differed between the two analysts, they were then compared and discussed until there was joint agreement. After the final thematic coding was completed, illustrative quotes were selected.

## Results

### Substance Use

Participants were, overall, very stable on their medication for OUD, reflected in their high levels of abstinence from opioids and other substances at baseline. Over the first three months of study involvement, these high rates of abstinence from opioids and overall substance use were maintained for both study groups, and there was no difference between study groups on substance use during this time frame (see Table 3).

Similarly, high rates of abstinence from other substances were seen in both groups and both groups improved slightly over time. Our pre-specified definition of overall substance use did not include cannabis use, which was by far the most frequently used substance assessed, followed by methamphetamines (Table 3). Other substances were rarely used during the study period.

### Mental Health Distress

PTSD symptoms significantly improved for those who received MABT+MOUD compared to those in MOUD (*p* = 0.02). Depression and anxiety symptoms did not show a significant between group difference (see Table 4).

All mental health outcomes improved for both study groups across the three-month study time-period (see Table 4), and there was a concomitant decrease in the proportion of participants who scored above the screening cut-points on each mental health symptom measure (PTSD, Depression, Anxiety). Notably, the drop from above to below the cut-off for moderate anxiety was significantly greater for MABT+ MOUD (42% to 19%) vs. MOUD (38% to 31%), *p* = 0.01.

### Emotion Regulation Difficulties

Emotion regulation difficulties showed a greater reduction for MABT+MOUD than for those in MOUD but did not reach significance on the total score (*p* = 0.07) (Table 4), or on any of the subscales. Notably, when gender was included in the model, the results showed a significant 3-way interaction (Treatment group × Month × Gender) indicating a greater increase in goal-directed behavior (on the DERS) among women in MABT + MOUD vs. MOUD only vs. men in MABT+ MOUD vs. MOUD (*p* = 0.02).

### Pain Severity, Pain Interference and Physical Symptom Frequency

Pain severity significantly improved for those in MABT compared to MOUD (*p* = 0.04). The activity pain interference score also showed significant improvement for those in MABT+MOUD than those in MOUD (*p* = 0.02), see Table 4. The affective pain interference score did not show a between-group difference. A clinically meaningful mean reduction (≥ 1 point) in overall pain interference was evident among both study groups: in 53 (39%) of MABT participants, and in 41 (31%) of MOUD participants.

Participants in both groups reported an average of 8 different physical symptoms at baseline. The most common among these were muscle aches, back aches, numbness or tingling, and waking with stiff or swollen joints (occurring “often”, or “always/almost always” by 50% of the total sample). Other highly endorsed frequent symptoms were ones potentially associated with taking buprenorphine/methadone such as excessive sweating, constipation/diarrhea, and feeling tired even when well rested. The total frequency of physical symptoms decreased significantly for MABT+ MOUD compared to MOUD (*p* = 0.002). We looked post-hoc at the subset of symptoms associated with MOUD listed above, and these also decreased significantly for MABT+MOUD vs. MOUD (*p* = 0.02), see Table 4.

### Interoceptive and Mindfulness Skills

Interoceptive awareness significantly improved for MABT+MOUD vs. MOUD (*p* < 0.001), see Table 4. All MAIA scales showed significant improvement, with the exception of the two scales *Not Distracting* and *Not Worrying* (see Table 5). The *Not Distracting* and *Not Worrying* scales were designed specifically to assess the use of interoceptive skills to manage coping with pain; the findings were similar when comparing study groups in the full sample and when comparing groups among only those with chronic pain.

Mindfulness skills showed greater improvement for those who received MABT but not at the level of significance (*p* = 0.07), see Table 4. When gender was included in the model, the results showed a significant 3-way interaction (Treatment group × Month × Gender) indicating a greater increase in mindfulness skills for women in MABT+MOUD vs. MOUD compared to men in MABT+MOUD vs. MOUD (*p* = 0.002).

### Opioid Craving

The overall sample craving level was low, with a mean level of 1.6 on an 11-point scale, see Table 4. There was no between-group difference in change in craving level over time, *p* =0.18, see Table 4. Approximately 46% of the total sample endorsed craving for opioid medication (48% in MABT+MOUD; 43% in MOUD) at baseline, with a mean craving level at 4.2 among this subgroup. At three months the MABT+MOUD study group showed a reduction in endorsed craving of prescribed opioid medication (38%) whereas the MOUD group showed no change (43%). There was, however, no between group change in craving level in this subgroup see Table 4.

Craving for non-prescribed opioids was endorsed by 32% of the total sample (32% in MABT+MOUD; 32% in MOUD) at baseline, with a mean craving level at 4.2 among this subgroup. At three months, there was a slight drop in endorsed craving for non-prescribed opioids in both study groups (28% MABT+MOUD; 27% MOUD); there was no significant between-group difference over time on craving level for non-prescribed opioids, see Table 4.

### Intervention Experience

Five primary themes emerged from the participants’ descriptions of their MABT intervention experience. Each theme is listed below with a brief synopsis; please see Table 6 for example quotes.

#### Increased Awareness and Acceptance:

Participants gained awareness of their physical sensations and their emotions, and responses highlighted an increased awareness of the link between physical sensations and emotional experience (for example, feeling more pain when stressed). In addition, the theme of acceptance often emerged in conjunction with awareness, suggesting increased comfort with noticing and attending to physical sensations and emotions.

#### Increased Self Care and Self-Agency:

Participants felt motivated to engage in self-care activities and experienced a sense of increased self-agency as they made new and healthier choices about how to spend their time.

#### Reduced Symptomatic Distress:

Participants experienced reduced symptomatic distress, both physical and emotional. The importance of home practice for integrating newly learned skills into daily life was often mentioned.

#### Improved Emotion Regulation:

Participants learned to emotionally regulate when stressed; to help manage mental health symptoms, and to help manage physical pain. They often identified specific interoceptive skills that were applied to help emotionally regulate.

#### MABT Facilitated OUD Treatment/Recovery:

Interoceptive sensibility was identified as a having a role in recovery, involving many of the themes above that together brought home the importance of one’s body/self as something worth caring for.

### MABT Take Home Practice

Take-home practice logs were used to record participant reported use of MABT take-home practice each week during the intervention period. Participants reported practicing an average of 4.5 days per week for an average of 13.7 minutes per day.

### Adverse Events

Adverse events were collected at each assessment time frame for the prior 3-month period, as well as weekly by the interventionists for participants assigned to MABT. In this initial pre-post study timeframe, there were no serious adverse events reported.

## Discussion

These immediate pre-post intervention findings of a randomized trial of Mindful Awareness in Body Oriented Therapy (MABT) + MOUD vs. MOUD found high rates of days abstinence in both groups, with no significant differences between groups from baseline to 3 months. This was a MOUD treatment-responsive sample, demonstrated by their low baseline levels of use and the average duration of over a year in MOUD treatment. While stable from a substance use perspective, participants in this study were experiencing high rates of co-occurring chronic pain and mental health symptoms, including PTSD, depression, and anxiety. Thus, the findings from this study highlight the response to MABT on secondary outcomes with MABT + MOUD outperforming those in MOUD on PTSD symptoms, pain severity, pain interference on activities, frequency of physical symptoms, and interoceptive awareness. Patient reports of their experiences in MABT highlight important domains of improvement, some of which dove-tail with findings of significant between-group positive change on health outcomes such as symptomatic distress, and interoceptive sensibility. Other qualitative themes point to important constructs not otherwise measured such as self-agency that may be critical to understanding processes underlying improved health outcomes and facilitating MOUD treatment recovery goals.

PTSD symptoms were significantly improved among those who received MABT + MABT compared to MOUD, and in addition there was a significant drop from above to below the cut-off for moderate anxiety. The lifetime trauma experiences among this sample were extensive (Rodriguez et al., 2024). The MABT protocol is designed to support and build the capacity for deep attentional presence in the body. The process of engaging in compassionate and sustained mindful attention to inner body experience during MABT sessions often led to the emergence of increased awareness and insight related to past traumatic experiences and current anxious responses to life events. These experiential insights were often accompanied by participant recognition that attending to and processing trauma was a critical component for their continued substance use recovery (see related example in Table 6 specific to theme of trauma recovery). The significant improvement in PTSD symptoms in response to MABT is notably stronger than in prior MABT SUD treatment studies (Price et al., 2019; Price, Wells, Donovan, & Rue, 2012), and may be due to the higher level of treatment stability in this sample, involving more capacity for engagement in interoceptive experience and reappraisal processes.

MABT recipients demonstrated a significantly greater reduction in pain severity compared to MOUD alone. This reduction in pain severity aligns with the concomitant significant reduction in frequency of physical symptoms among those who received MABT compared to those that did not. The qualitative outcomes also highlighted reduced physical symptoms and pain. The significant improvement in activity-based pain interference highlights other more functional improvements in pain due to the intervention. These pain-related findings are similar to a recent mindfulness-based treatment that was delivered solely to patients with chronic pain on long-term opioid therapy (N=230) that also found significant reductions in pain severity and interference (Garland et al., 2024). Interoceptive awareness training is an iterative process requiring the interplay between subjective perception of and attention to internal bodily sensations and the cognitive-affective appraisal of these bodily sensations that addresses the mind-body interplay inherent to chronic pain and underlying self-regulation processes fundamental to the treatment of chronic pain (Price & Mehling, 2016). The significant positive pain and physical symptom outcomes seen in this study of interoceptive training through MABT, aligns with the recognition that interoception is a critical component that needs to be addressed in the treatment of chronic pain conditions (Bonaz et al., 2021) and research highlighting self-regulation of sensory and affective experiences as candidate mechanisms for promoting long-lasting reductions in pain and corresponding comorbidities ^(McCracken & Turk, 2002; Villemure & Bushnell, 2002)^.

Among those that received MABT vs. MOUD alone, only women showed significant improvement in response to any aspect of the DERS, specifically goal-directed behavior which is the ability to concentrate or get things done when upset. This gender specific finding dovetails with the significant improvement in mindfulness skills seen only among women (vs men) in response to MABT. These findings raise questions about whether women compared to men gained more capacity to manage challenging emotions possibly through gaining a shift in somatic reappraisal through increased mindfulness skills, and points to possible gender-based differences in response to mindfulness training (Rojiani et al., 2017).

Overall, however, men and women were equivalent in their response to MABT and to the interoceptive skills learned, as evident in the highly significant increases in interoceptive skills among those who received MABT+MOUD compared to those in MOUD. MABT facilitates participant development of sensory and emotional awareness through attention to sensory cues as well as through skills training to sustain mindful interoceptive practice to promote insight and somatic reappraisal processes understood to promote behavior change (Price & Weng, 2021). The many scales of the MAIA, particularly those that address using interoceptive skills to increase emotional awareness, regulate if stressed, and increase self-understanding (*Emotional Awareness Scale, Self-Regulation Scale and Body Listening Scale*) highlight the regulatory skills learned by those who received MABT. The qualitative evaluation of the subjective experience of the participants, important for understanding the experience and impact of an intervention, mirror many of these MAIA scale constructs. For many participants the use of mindful interoceptive awareness was new and unfamiliar, yet the positive response to this intervention reflected in the high completion rate, daily engagement in take home practice, and perceived helpfulness for OUD recovery, speak to the high implementation feasibility and perceived helpfulness of this approach as an adjunct to MOUD.

The results of this study are both similar and distinct from other studies to date examining the benefits of mindfulness-based approaches as an adjunct to MOUD, and for those using or misusing prescribed opioids (Cooperman et al., 2023; Garland, et al., 2022; Garland, et al., 2023; Garland et al., 2024). The MORE and MABT interventions have overlapping components and are both focused on developing mindfulness to promote reappraisal processes that support health and well-being. These intervention approaches are distinct in their delivery (group for MORE; individual and with the use of touch for MABT) as well as in their psychoeducational focus (learning savoring practices and cognitive reappraisal of maladaptive thoughts in MORE; learning practices to support sustained interoceptive attention and related reappraisal of somatic and emotional experiences in MABT). Like MABT, the MORE studies have documented a range of positive mental health, pain severity and pain-related functional outcomes, highlighting health outcome improvements that are possible in response to mindfulness-based interventions with this population. In contrast to our study, the MORE studies focused specifically on populations with chronic pain and were able to show improvements in depression symptoms, which are highly comorbid with chronic pain. We found no immediate post intervention improvements in depression symptoms in our study sample, where just over half of the participants reported chronic pain. Our study of MABT, in contrast to MORE trials, found improvements in measures of anxiety and PTSD. Of the MORE trials, the one most similar to the current study is with patients in methadone maintenance but with higher baseline levels of drug use (Cooperman et al., 2023). The benefits of MABT on substance use frequency may become apparent in our longitudinal follow-up assessments or may require testing in populations with higher levels of substance use at baseline.

### Limitations and Future Directions

Limitations of this study include those inherent to the application of a new treatment modality in complex care settings. We included urban and rural MOUD patients receiving buprenorphine or methadone in primary care and specialty addiction clinics, reflecting different baseline clinical characteristics and requiring careful attention to our analytic procedures. Given the wide array of outcome measures and the variable patterns of co-occurring medical and mental health disorders, demonstrating improvement over a large sample with a diverse set of clinical issues can be challenging. Because we chose to enroll patients after they had stabilized on medication to maximize their ability to participate in and benefit from the intervention, we had less opportunity to demonstrate improved substance use than in an intervention applied immediately on treatment initiation, when patients are at highest risk of drop-out. Also, this study collected substance use data via retrospective self-report using the TLFB interview and did not include verification via biochemical drug screens. Last, we provided a $10 grocery store gift card for completion of each MABT session, limiting generalizability to real-world settings where this would not be done. Future study to examine MABT specifically for people in early treatment would enhance the ability to evaluate intervention impact on substance use in this population. Likewise, examining MABT in early treatment when there are more frequent medical visits would allow for easier verification of self-reported drug use from electronic medical records data.

Strengths of this study include a randomized study design and a large sample treated in diverse settings. There were few overall eligibility restrictions for study enrollment which increases generalizability of findings to real-world settings. We assessed a wide range of clinical outcomes that mirror the complexity of patients receiving and benefitting from MOUD. Our ability to recruit participants and provide a substantial dose of MABT demonstrates MOUD patient interest in, and willingness and ability to take further steps in their recovery and engage in an unfamiliar integrative health approach. The interventionists delivering this intervention were licensed massage therapists who had, at most, a Bachelors or Masters-level education which may contribute to dissemination efforts particularly in rural treatment settings.

In conclusion, this study shows MABT training to be efficacious for immediate pre-post intervention improvements of PTSD symptoms, pain severity, pain activity interference, physical symptom frequency, and interoceptive awareness among people stabilized on medication in MOUD. This study was the first full-scale randomized controlled trial focused on interoceptive training as an adjunct to MOUD. The significant improvements in interoceptive sensibility and concomitant improvements in health outcomes, are consistent with neurocognitive models that link interoception to health outcomes (Quadt, Critchley & Garfinkel, 2018), important for MOUD treatment. The overall ease of recruitment and high participant engagement points to the future promise of implementing MABT as an integrative approach within the context of community treatment for MOUD.

## Figures and Tables

**Table 1.** Demographic and Clinical Characteristics

	Total	TAU	MABT

N	303	148	155
Age, median (range)	40 (21–73)	41 (22–71)	37 (21–73)
Gender Identity			
Male	144 (48%)	69 (47%)	75 (48%)
Female	157 (52%)	78 (53%)	79 (51%)
Non-binary	2 (1%)	1 (1%)	1 (1%)
Hispanic	27 (9%)	15 (10%)	12 (8%)
Race			
White	238 (79%)	113 (76%)	125 (81%)
More than one race	28 (9%)	19 (13%)	9 (6%)
Black or African American	16 (5%)	7 (5%)	9 (6%
Native American	13 (4%)	4 (3%)	9 (6%)
Asian	3 (1%)	2 (1%)	1 (1%)
Hawaiian or Pacific Islander	4 (1%)	3 (2%)	1 (1%)
Marital Status			
Married or Domestic Partnership	51 (17%)	27 (18%)	24 (15%)
Single	215 (71%)	97 (66%)	118 (76%)
Unknown (Endorsed “Other”)	36 (12%)	23 (16%)	13 (8%)
Highest Education Level			
11th grade or less	36 (12%)	15 (10%)	21 (14%)
High school or GED	132 (44%)	65 (44%)	67 (43%)
Two-year college/technical school	103 (34%)	55 (37%)	48 (31%)
College degree (e.g., BA, BS)	32 (11%)	13 (9%)	19 (13%)
Monthly Income			
No monthly income	47 (16%)	27 (18%)	20 (13%)
Some income but less than $1000	132 (44%)	64 (43%)	68 (44%)
$1000 or more	124 (41%)	57 (39%)	67 (43%)
Employed	104 (35%)	47 (31%)	57 (37%)
Full-time	69 (23%)	33 (22%)	36 (23%)
Part-time	35 (12%)	14 (9%)	21 (14%)
Insurance^a^			
Medicaid	219 (72%)	107 (72%)	112 (72%)
Medicare	69 (23%)	36 (24%)	33 (21%)
Private	36 (12%)	13 (9%)	23 (15%)
None	5 (2%)	4 (3%)	1 (1%)
Chronic Pain 3 Months or More	172 (57%)	87 (59%)	85 (55%)
Received Mental Health Care in Past 3 Months	141 (47%)	66 (45%)	75 (48%)
Above Cut-off Mental Health Disorder			
Post Traumatic Stress Disorder (PTSD)^b^	124 (41%)	62 (42%)	62 (40%)
Moderate Depression^c^	147 (49%)	70 (47%)	77 (48%)
Moderate Anxiety^d^	121 (40%)	56 (38%)	65 (41%)
Lifetime Trauma Exposure (TLEQ)			
Childhood Sexual Abuse	165 (54%)	86 (58%)	79 (51%)
Childhood Physical Abuse	127 (42%)	66 (45%)	61 (40%)
Adult Sexual Assault	110 (37%)	54 (36%)	56 (37%)
Adult Physical Assault by Stranger	163 (54%)	84 (57%)	79 (51%)
Intimate Partner Violence (IPV)	223 (74%)	113 (80%)	110 (77%)
Accidents/Non-interpersonal Trauma	243 (86%)	117 (82%)	126 (89%)
Medication for Opioid Use Disorder (MOUD)			
Methadone	35 (12%)	17 (12%)	18 (13%)
Buprenorphine	249 (88%)	125 (88%)	124 (87%)
Time in Treatment Prior to Enrollment			
3–6 months	25 (9%)	10 (7%)	15 (11%)
6–12 months	44 (15%)	16 (11%)	28 (20%)
> 12 months	188 (66%)	100 (70%)	88 (62%)

*Note:* Values are number (percentage) unless otherwise indicated.

a Respondents could select multiple responses.

b PCL-5 ≥ 31

c PHQ-9 ≥ 10

d GAD-7 ≥ 10

**Table 2.** Components of MABT

Stage 1 (Sessions 1–2)	Stage 2 (Sessions 3–4)	Stage 3 (Sessions 5–8)

Check-in (20)	Check-in (20)	Check-in (20)
Body Literacy (40)	Body Literacy (10)	Body Literacy (10)
	Interoceptive Awareness Training (30)	Mindful Body Awareness Practice (30)
Session Review (15)	Session Review (15)	Session Review (15)
Take Home Practice	Take Home Practice	Take Home Practice

*Note.* Values in parentheses represent time spent in number of minutes in each session.

**Table 3.** Substance Use Outcomes and Assessed Substances

	MABT+ MOUD		MOUD		Group × Time	
	
Construct (Scale)	Baseline	3 Months	Baseline	3 Months	χ^2^	*p*

N	155	139	148	139		

***Primary Outcomes***	Mean (SE)	Mean (SE)	Mean (SE)	Mean (SE)		
% Days Abstinent Opioid Use^a^	96.9 (0.9)	97.1 (1.0)	96.8 (1.1)	97.9 (1.0)	0.44	0.50

% Days Abstinent Total Substance Use^a,b^	89.1 (1.9)	90.7 (1.9)	88.6 (2.1)	91.4 (1.8)	0.54	0.46

***Assessed Substances***	Mean (SD)	Mean (SD)	Mean (SD)	Mean (SD)		
% Days Abstinent Alcohol Use	93.6 (18.3)	95.4 (14.2)	92.5 (20.3)	93.0 (19.0)		

% Days Abstinent Heavy Drinking^c^	98.6 (9.1)	99.7 (1.6)	99.1 (4.9)	99.4 (3.5)		

% Days Abstinent Amphetamines	100.0 (0.2)	99.9 (0.5)	99.9 (0.6)	99.9 (0.5)		

% Days Abstinent Barbiturates	100.0 (0.1)	100.0 (0.0)	100.0 (0.0)	100.0 (0.0)		

% Days Abstinent Benzodiazepines	98.7 (7.1)	98.6 (7.3)	99.4 (5.3)	99.1 (8.1)		

% Days Abstinent Cannabis	70.8 (39.6)	72.6 (37.8)	74.0 (40.9)	75.0 (40.5)		

% Days Abstinent Cocaine	99.5 (2.6)	99.3 (3.5)	95.6 (17.4)	96.6 (15.6)		

% Days Abstinent Hallucinogens	99.9 (0.5)	99.8 (1.4)	99.9 (0.5)	100.0 (0.2)		

% Days Abstinent Methamphetamines	92.6 (21.2)	94.5 (18.7)	92.3 (23.4)	94.5 (18.7)		

a Data are estimates from a GEE model with a logit link and adjusted for number of days since last assessment.

b Total Substance Use includes all unprescribed substances and heavy drinking days but does not include cannabis.

**Table 4.** Secondary Outcomes: Linear Mixed Model Results

	MABT + MOUD		MOUD		Group × Time	
Construct	Baseline	3 Months	Baseline	3 Months	χ^2^	*p*
N	155	139	148	139		
**Mental Health Distress and Coping**	Mean (SD)	Mean (SD)	Mean (SD)	Mean (SD)		
PTSD Symptoms^a^	27.7 (1.3)	21.6 (1.3)	27.7 (1.5)	25.2 (1.7)	5.62	0.02
Depression Symptoms^b^	9.9 (0.5)	7.9 (0.4)	9.9 (0.5)	8.5 (0.5)	0.93	0.34
Anxiety Symptoms^c^	8.7 (0.4)	6.3 (0.4)	8.7 (0.5)	7.3 (0.5)	2.79	0.10
Emotion Regulation Difficulties^d^	39.5 (0.9)	35.6 (0.9)	39.9 (1.1)	38.0 (1.2)	3.30	0.07
**Pain and Physical Symptoms**						
Severity ^e^	3.8 (0.2)	3.3 (0.2)	3.8 (0.2)	3.6 (0.2)	4.07	0.04
Interference – Activity ^e^	11.2 (0.7)	9.0 (0.7)	10.8 (0.7)	10.7 (0.7)	5.45	0.02
Interference – Affective ^e^	14.5 (0.8)	12.3 (0.8)	14.8 (0.9)	13.7 (0.9)	0.96	0.33
Physical Symptom Frequency ^f^	2.2 (0.04)	2.0 (0.04)	2.2 (0.04)	2.1 (0.05)	9.26	.002
**Interoceptive and Mindfulness Skills**						
Interoceptive Awareness (Total) ^g^	2.5 (0.1)	2.9 (0.1)	2.6 (0.1)	2.7 (0.1)	15.21	<0.001
Mindfulness Skills^h^	34.4 (0.7)	38.3 (0.6)	35.1 (0.7)	37.5 (0.7)	3.26	0.07
**Opioid Craving Level**						
Prescribed Opioids: Full Sample	1.7 (0.2)	1.2 (0.2)	1.5 (0.2)	1.3 (0.2)	1.82	0.18
Prescribed Opioids: Subsample ^i^						
Non-Prescribed Opioids: Subsample ^j^	4.2 (2.0)	4.4 (2.8)	4.0 (2.5)	4.6 (2.4)	0.25	0.62

a PHQ-9

b GAD-7

c PCL-5

d DERS-SF

e BPI

f MSC

g MAIA

h FMI

i Sample size at baseline, 3 months: MABT+MOUD (49, 34); MOUD (44, 34)

j Sample size at baseline, 3 months: MABT+MOUD (49, 36); MOUD (46, 34)

**Table 5.** MAIA Scales

	MABT		TAU		Group × Time	
Construct (Scale)	Baseline	3 Months	Baseline	3 Months	χ^2^	*p*
N	155	139	148	139		
**Interoceptive Awareness**	Mean (SE)	Mean (SE)	Mean (SE)	Mean (SE)		
Noticing	3.0 (0.1)	3.5 (0.1)	3.0 (0.1)	3.1 (0.1)	5.74	0.02
Not Distracting	1.9 (0.1)	2.3 (0.1)	1.8 (0.1)	2.0 (0.1)	0.84	0.36
Not Worrying	2.8 (0.1)	3.1 (0.1)	2.7 (0.1)	2.9 (0.1)	0.27	0.61
Attention Regulation	2.4 (0.1)	3.0 (0.1)	2.5 (0.1)	2.6 (0.1)	9.43	0.002
Emotional Awareness	3.1 (0.1)	3.5 (0.1)	3.3 (0.1)	3.3 (0.1)	8.18	0.004
Self-Regulation	2.4 (0.1)	3.3 (0.1)	2.6 (0.1)	2.8 (0.1)	15.16	<0.001
Body Listening	1.7 (0.1)	2.8 (0.1)	1.9 (0.1)	2.1 (0.1)	30.26	<0.001
Body Trusting	2.7 (0.1)	3.5 (0.1)	2.9 (0.1)	3.1 (0.1)	12.85	<0.001

MABT: Mindful Awareness in Body-oriented Therapy; TAU: Treatment as Usual. Data are estimates from linear mixed models.

**Table 6.** Themes and Exemplary Quotes Reflecting MABT Intervention Experience

Themes	Example Quote(s)
**Increased Awareness and Acceptance**	*“One thing that I have learned through MABT is to give myself grace, and that its okay to feel stressed or overwhelmed. That, instead of running from it, to just sit with it and accept the hard things that are going on and that I have a lot on my plate. I’ve learned to really accept my emotions more and just be with them verses trying to bottle them all up. I’ve also learned that certain physical pains in my body sometimes can have an emotional connection to them as well.”*
**Increased Self Care and Agency**	*“I am making decisions for myself, like getting my hair cut. I am putting on make-up and taking time for myself. Taking a walk or a drive to have time to myself. Listen to praise and worship music.”*
**Reduced Symptomatic Distress**	*“I don’t have feet pain anymore. Taking what I learned home has helped a tremendous amount to relieve the pain. It is so good to be able to walk around. I’m glad I got to learn.”* *“MABT is a beautiful thing and it has genuinely given/taught me tools that I can use lifelong. Because of my lupus diagnosis I have been suffering with Severe Panic attacks & MABT tools such as scanning and breathing etc. are the only things that help me either end a panic attack or prevent it.”*
**Improved Emotion Regulation**	*“I have anxiety, and am diagnosed with anxiety and depression. On my own now, I regulate my emotions instead of reacting to things or situations. To increase body awareness I do a quick body scan. I do this every day at least for 5 minutes. My emotions have become more balanced and less up and down all day. I can close my eyes in the morning when I wake up and think about a certain area and place my awareness in that and it helps me relax in the morning and whenever I need it. I can do it sometimes during groups at the clinic and it helps me relax.”*
**MABT Facilitated OUD Treatment/Recovery**	*“MABT helped me concentrate on my body more than the drugs and it made me feel connected to my body and keep it sober and it made me want to nurture my body and care about it more and give it the things it needs.”* *“Learning to connect with what my body is telling me has helped strengthen my recovery process and taught me how to better cope with certain things during stressful times. I was also able to work through some past traumas during the MABT sessions which I feel also has a first-hand connection with my recovery process. Having a safe place to open up about things on my mind at that moment and work through things that were going on in life at that time I feel like also lowers my chance of relapse.”*

## Data Availability

A de-identified data set is available through ICPSR at https://www.openicpsr.org/openicpsr/project/205381/version/V1/view
